# Deep-branching magnetotactic bacteria form intracellular carbonates enriched in trace metals

**DOI:** 10.1128/msystems.01131-25

**Published:** 2025-11-12

**Authors:** Peiyu Liu, Rongrong Zhang, Fanqi Meng, Chaoqun Zhang, Andrew P. Roberts, Yinzhao Wang, Kelei Zhu, Zhaoyang Cao, Yongxin Pan, Jinhua Li

**Affiliations:** 1 Key Laboratory of Deep Petroleum Intelligent Exploration and Development, Center for Oil-Gas Theories and Methods, Institute of Geology and Geophysics, Chinese Academy of Sciences, Beijing, China; 2 Laboratory for Marine Geology, Qingdao Marine Science and Technology Center, Qingdao, China; 3 Southern Marine Science and Engineering Guangdong Laboratory 590852 , Zhuhai, Guangdong, China; 4 College of Earth and Planetary Sciences, University of Chinese Academy of Sciences, Beijing, China; 5 School of Materials Science and Engineering, Peking University 12465, Beijing, Beijing, China; 6 Research School of Earth Sciences, Australian National University 2219, Canberra, Australian Capital Territory, Australia; 7 State Key Laboratory of Microbial Metabolism, School of Life Sciences and Biotechnology, Shanghai Jiao Tong University 12474, Shanghai, Shanghai, China; 8 Key Laboratory of Earth and Planetary Physics, Institute of Geology and Geophysics, Innovation Academy for Earth Sciences 74519, Beijing, Beijing, China; NYU Langone Health, New York, New York, USA

**Keywords:** intracellular calcification, magnetotactic bacteria, carbonate inclusions, heavy metal elements, biogeochemical cycling

## Abstract

Microbial biomineralization is a fundamental driver of global biogeochemical cycles, yet the ability of prokaryotes to form intracellular carbonates remains rarely documented. Here, we report three ecotypes of magnetotactic bacteria (MTB) affiliated with the *Pseudomonadota* and the deep-branching *Nitrospirota* phyla that concurrently synthesize magnetite magnetosomes and intracellular calcium carbonate inclusions enriched in Ba, Mg, and Ni. These carbonate granules are typically spherical and contrast with the highly ordered morphology of magnetite crystals. Comparative genomic analyses reveal that these MTB encode multiple metal-permease systems (e.g., GDT1, CorA, ZnuA2), which suggests both a capacity for selective uptake of divalent cations from their environment and a process likely linked to intracellular carbonate precipitation. By uncovering new examples of bacterial intracellular calcification, our findings expand the known diversity and genetic basis of prokaryotic biomineralization. Moreover, they highlight a potential role of MTB in mediating heavy-metal cycling and provide a refined framework for understanding microbially driven carbonate formation.

**IMPORTANCE:**

Intracellular biomineralization is a hallmark of animals and algae, yet among prokaryotes, it has traditionally been associated with a limited range of lineages and minerals. This study reveals that magnetotactic bacteria (MTB) from both the *Pseudomonadota* and the deep-branching *Nitrospirota* phyla are capable of intracellularly forming carbonate granules enriched in diverse divalent cations, including environmentally scarce trace metals Ba²⁺ and Ni²⁺, and biologically essential Mg²⁺. These findings significantly expand the known taxonomic and functional diversity of prokaryotic intracellular calcifiers. By integrating electron microscopy, metagenomics, and structural protein modeling, we propose a potential metal-selective transport system that facilitates trace element accumulation and carbonate precipitation. This work establishes a previously underappreciated role for MTB in trace metal biogeochemical cycling (i.e., Ba²⁺ and Ni²⁺) and suggests that intracellular calcification may be a more widespread bacterial trait than previously assumed.

## INTRODUCTION

Biomineralization, the biologically mediated precipitation or transformation of minerals, is central to the global cycling of major elements (e.g., C, Si, O, Fe, S, N, Ca) (1–8). Although eukaryotes frequently implement highly controlled, intracellular mineralization pathways, prokaryotes are traditionally viewed as mediating mineral formation predominantly in the extracellular milieu, where diverse metabolic processes (e.g., urea hydrolysis, denitrification, and sulfate reduction) drive secondary precipitation (9, 10). Such microbially induced mineralization can also facilitate heavy metal sequestration by incorporating Pb^2+^, Cd^2+^, and Cu^2+^ into extracellular carbonates (11, 12).

Compared to widespread extracellular calcification in prokaryotes, intracellular carbonate biomineralization has long been regarded as rare. For example, large sulfur bacteria of the genus *Achromatium* harbor intracellular calcite inclusions up to ~5–6 µm in diameter (13, 14), whereas cyanobacteria contain amorphous sub-micron carbonates located either at cell poles or scattered throughout the cytoplasm (15–17). More recently, electron microscopy and synchrotron-based microspectroscopy advances reveal that certain *Alphaproteobacteria* and *Gammaproteobacteria* (*Pseudomonadota* phylum) magnetotactic bacteria (MTB) can also form micrometer to sub-micrometer intracellular carbonate granules (18–20), thereby broadening the phylogenetic distribution of bacterial calcifiers. Most reported carbonate inclusions are dominated by Ca, with occasional incorporation of Mg, Ba, or Sr (15, 19). Nevertheless, the biodiversity of intracellularly calcifying bacteria and the molecular mechanisms underlying metal accumulation remain poorly understood.

Here, we report three MTB ecotypes, including the first representatives from the deep-branching *Nitrospirota* phylum. In addition to Ca²^+^ and Mg²^+^, these MTB concentrate environmentally scarce Ba²^+^ and Ni²^+^ within intracellular carbonate granules. By combining high-resolution microscopy, metagenomics, and structural protein modeling, we identify candidate metal transporters (e.g., GDT1, CorA, ZnuA2) potentially involved in Ba/Ni incorporation. This work broadens the phylogenetic scope of bacterial intracellular carbonate formers and highlights MTB as a previously underappreciated player in heavy-metal (i.e., Ba²^+^ and Ni²^+^) biogeochemical cycling.

## RESULTS

### Aquatic chemistry and phylogenetic identification of intracellularly calcifying MTB

**Fig 1:**
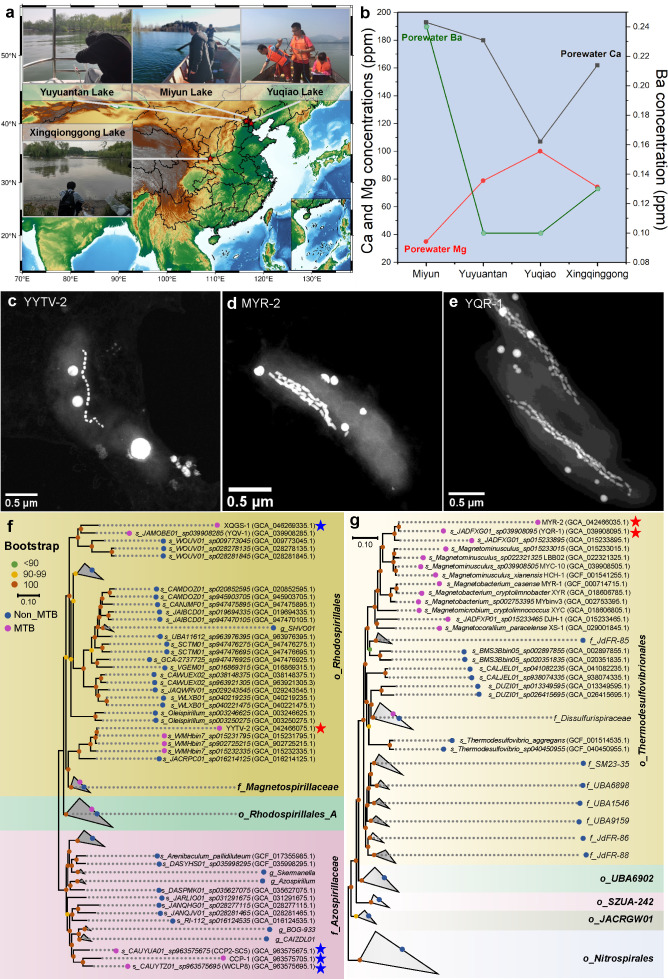
Habitat chemistry, cell morphology, and phylogeny of intracellularly calcifying MTB. (**a**) Location maps for the three freshwater lakes (Miyun, Yuyuantan, and Yuqiao) in northern China from which MTB were sampled. This map was designed using the Generic Mapping Tools software (https://www.generic-mapping-tools.org). (**b**) Measured ion concentrations (ppm) in the overlying sediment pore water at each site: Ca²^+^, Mg²^+^, Ba²^+^, and Ni²^+^ (Ni²^+^ was below detection limit [0.007 ppm] at all sites). (**c**–**e**) HAADF-STEM images of the three newly identified MTB strains (**c**) YYTV-2, (**d**) MYR-2, and (**e**) YQR-1, highlighting their intracellular carbonate granules (bright spheres). (**f**–**g**) Maximum-likelihood phylogenetic tree of the *Rhodospirillales* order (**f**) and the *Thermodesulfovibrionia* class (**g**) bacteria based on 120 single-copy ortholog genes. The *Azospirillaceae* family and *Nitrospirales* order genomes serve as the outgroup, respectively. Strains known to produce intracellular carbonates are marked with blue stars, and the calcifier characterized here is marked with a red star. Bootstrap values are shown at branch nodes as percentages based on 1,000 replicates. GenBank accession numbers are listed in parentheses. Detailed genomic information of bacteria selected for these trees is provided in Data S1 and S2**.**

We identified three uncultured MTB ecotypes (i.e., MYR-2, YYTV-2, YQR-1) from lakes Miyun (40°31′11.7″ N, 116°50′7.0″ E), Yuyuantan (40°24′12.1″ N, 116°24′43.2″ E), and Yuqiao (40°2′28″ N, 117°27′21″ E) in Northern China. These lakes are all weakly alkaline freshwater lakes with salinity ≤0.95‰, pH of 7.52–8.34, and metal cation concentrations in sediment pore water of 101–193 ppm Ca²^+^, 32–100 ppm Mg²^+^, 0.1–0.24 ppm Ba²^+^, and <0.007 ppm Ni²^+^ (Fig. 1a and b; Table 1).

**TABLE 1:** Sampling locations and environmental factors^*a*^

Sample site	Location	Water temp (°C)	pH	Salinity (‰)	Sediment pore water concentration (ppm)			Latitude (N)	Longitude (E)
					Ca^2+^	Mg^2+^	Ba^2+^		
Miyun Lake	Beijing	19	7.52	0.19	193	34.9	0.24	40°31′11.7″	116°50′7.0″
Yuqiao Lake	Tianjin	21	7.57	0.24	107	100	0.1	40°2′28″	117°27′21″
Yuyuantan Lake	Beijing	18	8.34	0.95	180	78.8	0.1	40°24′12.1″	116°24′43.2″
Xingqinggong Lake	Xi’an	22	7.93	0.55	162	74.3	0.13	34°15′49.8″	108°58′9.3″

^*a*^ Ni²^+^ concentrations in all lakes were lower than the detection limit (0.007 ppm).

The morphologies of MYR-2 and YYTV-2 cells were identified by the correlative fluorescence *in situ* hybridization and scanning electron microscopy (FISH-SEM) method, followed by transmission electron microscopy (TEM) observations (Fig. S1 to S4). YYTV-2 vibrio cells have an average length and width of 2.4 ± 0.3 µm and 0.75 ± 0.1 µm, respectively (*n* = 37). They produce 22 ± 3 cubo-octahedral magnetosomes per cell, with a mean length and width of 56.2 ± 12.3 nm and 41.2 ± 11.3 nm, respectively (*n* = 544; Fig. 1c). MYR-2 cells are curved rods with an average length and width of 2.8 ± 0.2 µm and 0.7 ± 0.1 µm, respectively (*n* = 26). These cells produce 56 ± 13 curved, bullet-shaped magnetosomes per cell, with a mean length and width of 90.2 ± 24.3 nm and 40 ± 3.6 nm, respectively (*n* = 532); these magnetosomes are organized into a chain along the concave side of the curved cell (Fig. 1d). The morphological characteristics of YQR-1 have been described in a previous study (Fig. 1e) (21).

Genome-based phylogenetic analysis placed YYTV-2 within the *Rhodospirillales* (*Pseudomonadota* phylum; Fig. 1f; Data S1) and both MYR-2 and YQR-1 within *Thermodesulfovibrionales* (*Nitrospirota* phylum; Fig. 1g; Data S2). Average nucleotide identity between MYR-2 and YQR-1 is ~99.8%, indicating that they represent the same species despite originating from sediments with different cation concentrations.

### Chemical characterization of MTB-formed intracellular carbonates

**Fig 2:**
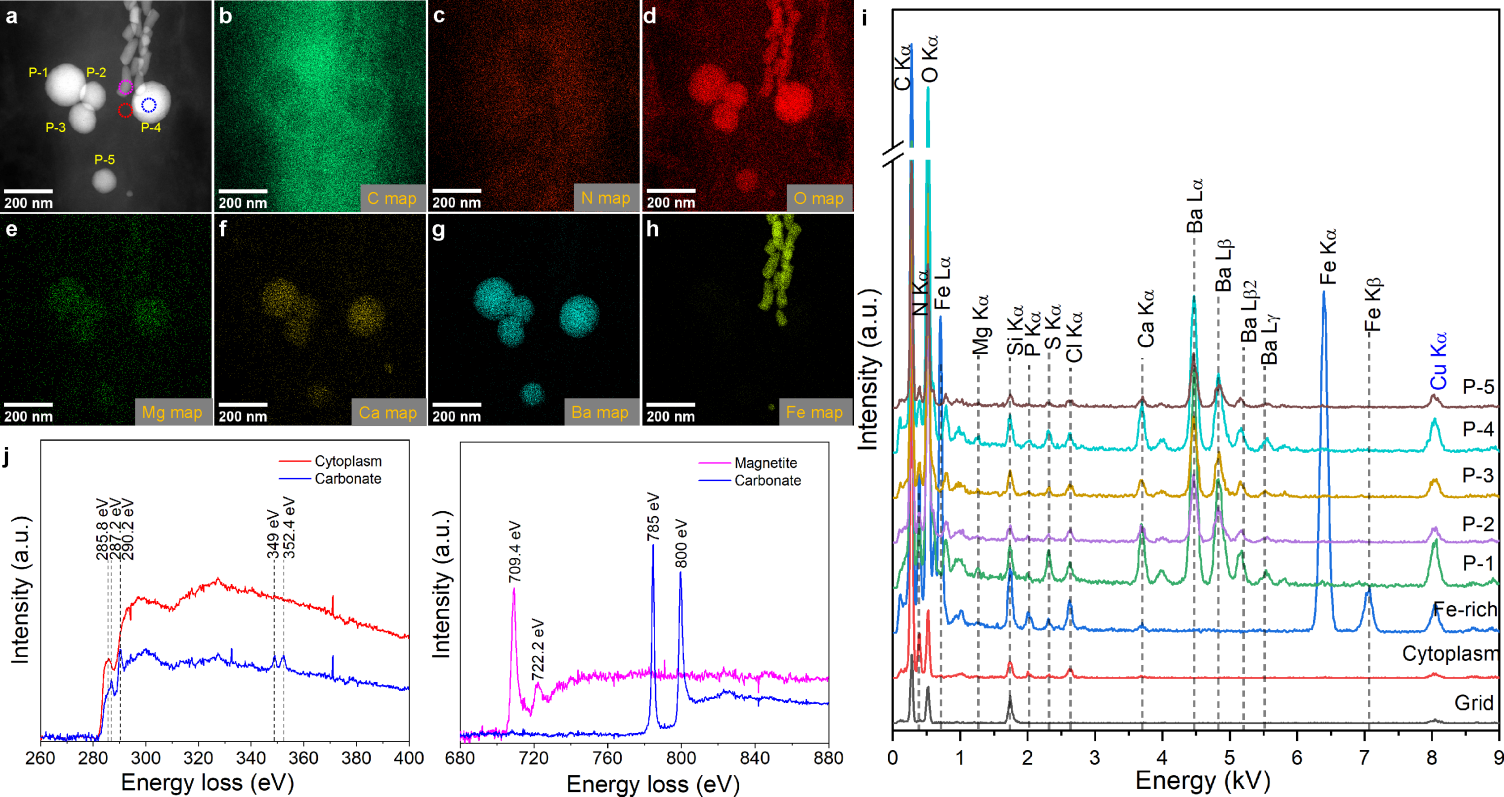
Chemical and mineralogical characterization of Ca–Ba–Mg carbonate inclusions in a MYR-2 cell. (**a**) HAADF-STEM image of five carbonate inclusions within a MYR-2 cell. (**b**–**h**) Corresponding STEM-EDXS elemental maps illustrating the distribution of (**b**) carbon (C Kα), (**c**) nitrogen (N Kα), (**d**) oxygen (O Kα), (**e**) magnesium (Mg Kα), (**f**) calcium (Ca Kα), (**g**) barium (Ba Lα), and (**h**) iron (Fe Kα). (**i**) STEM-EDX spectra from selected regions of interest (ROI), a carbonate, a magnetosome, the cytoplasm, and the TEM grid, with characteristic peaks for each component (a.u., arbitrary unit). (**j**) TEM-EELS spectra obtained at the C K-edge (280–320 eV), Ca *L*_2,3_-edge (340–360 eV), Fe *L*_2,3_-edge (700–730 eV), and Ba *M*_4,5_-edge (760–810 eV) for a magnetite magnetosome (carmine ROI), a carbonate inclusion (blue ROI), and cytoplasm (red ROI) as indicated by the dashed circles in panel **a**. These spectra match those of magnetite, calcium carbonate, and barium carbonate, respectively.

**Fig 3:**
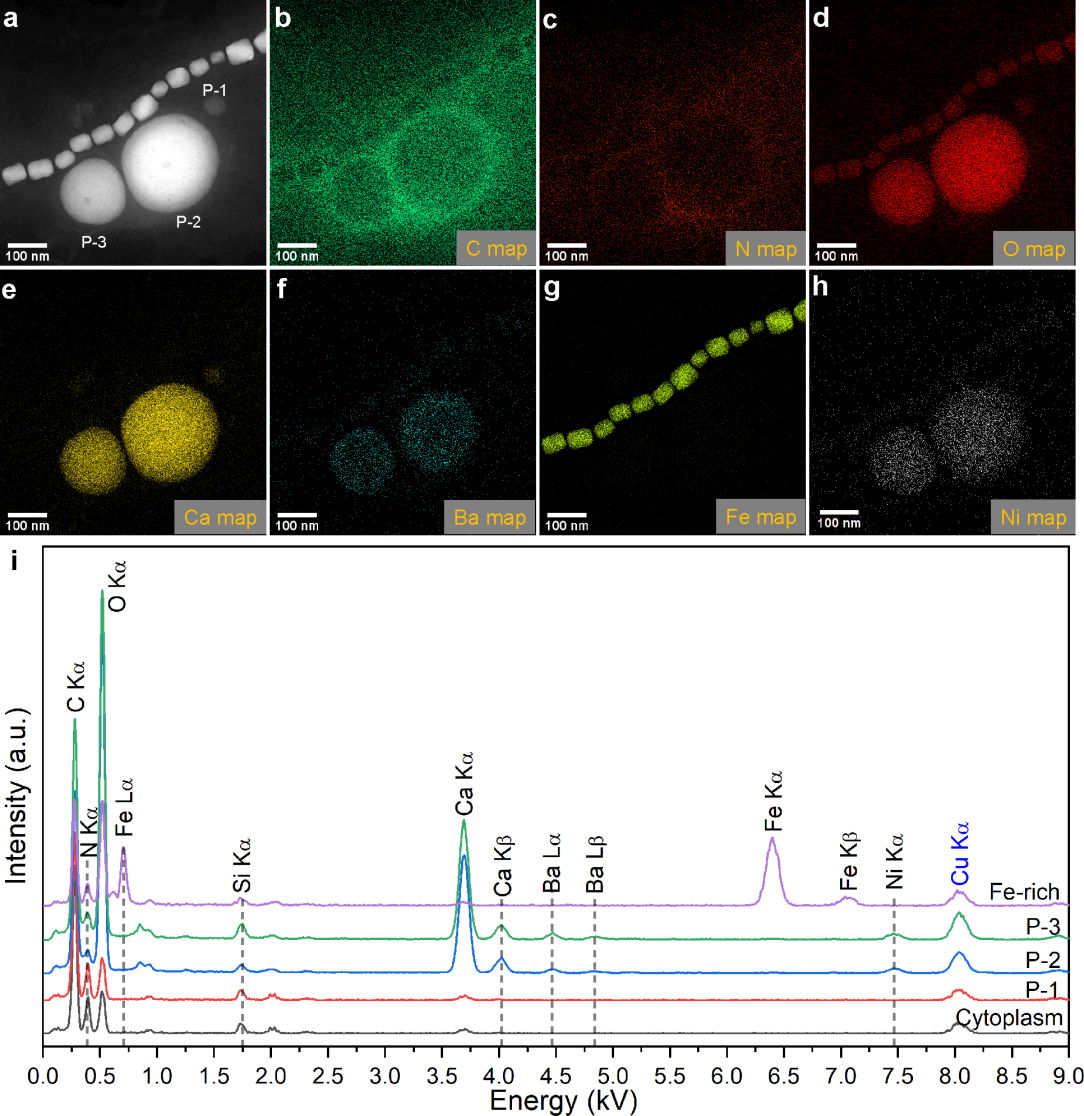
Chemical characterization of Ca–Ba–Ni carbonate inclusions in a YYTV-2 cell. (**a**) HAADF-STEM image of three distinct carbonate inclusions within a YYTV-2 cell, along with a magnetosome chain. (**b**–**h**) STEM-EDXS elemental maps of the same region, indicating the distribution of (**b**) carbon (C Kα), (**c**) nitrogen (N Kα), (**d**) oxygen (O Kα), (**e**) calcium (Ca Kα), (**f**) barium (Ba Lα), (**g**) iron (Fe Kα), and (**h**) nickel (Ni Kα). (**i**) Representative STEM-EDXS spectra collected from three regions of interest: magnetosome (Fe-rich), carbonate inclusions (P-1, P-2, P-3), and cytoplasm.

TEM observations reveal that all analyzed MYR-2, YQR-1, and YYTV-2 cells contain 1–7 spherical carbonate inclusions, with mean diameters of 0.11 ± 0.03 µm (*n* = 26), 0.13 ± 0.03 µm (*n* = 33), and 0.22 ± 0.08 µm (*n* = 29), respectively (Fig. 2 and 3; Fig. S3 to S9). Selected-area electron diffraction (SAED) patterns indicate that carbonate inclusions in YYTV-2 are amorphous, whereas those in MYR-2 have weak crystallinity (Fig. S3 and S4). High-angle annular dark-field scanning TEM (HAADF-STEM), combined with energy dispersive X-ray spectroscopy (EDXS) and electron energy-loss spectroscopy (EELS), reveals that all three ecotypes predominantly incorporate Ca, with frequent Ba, Mg, or Ni occurrences. Specifically, Ba is detected in all carbonate inclusions (*n* = 23) in MYR-2 and (*n* = 7) YQR-1, and ~44.4% of inclusions (*n* = 9) in YYTV-2. Ni appears in ~55.6% of YYTV-2 granules, whereas Mg occurs in ~47.8% of MYR-2 granules. Semi-quantitative STEM-EDXS mapping yields Ba/Ca ratios as high as ~20.4 in individual inclusions (Table S1). On average, Ba/Ca is 6.92 ± 1.65 (*n* = 5) for MYR-2, 8.37 ± 7.4 (*n* = 7) for YQR-1, and 0.05 ± 0.04 (*n* = 9) for YYTV-2. In contrast, the previously characterized ecotype XQGS-1 lacks detectable Ba in its carbonate inclusions (*n* = 10) (18). These observations suggest that each MTB ecotype regulates its carbonate composition both inter- and intra-species.

Although environmental Ba^2+^ and Ni^2+^ concentrations are extremely low (<0.24 and <0.007 ppm, respectively), MTB cells concentrate these metals to levels sufficient for Ba–Ni carbonate formation. Moreover, MYR-2 and YQR-1, despite differing ambient Ca^2+^ concentrations (193 vs 107 ppm, respectively), have similar Ba/Ca ratios in their carbonate inclusions. This demonstrates active cation selection and internal regulation, consistent with biologically controlled biomineralization processes.

### Genomic insights into heavy metal accumulation

**Fig 4:**
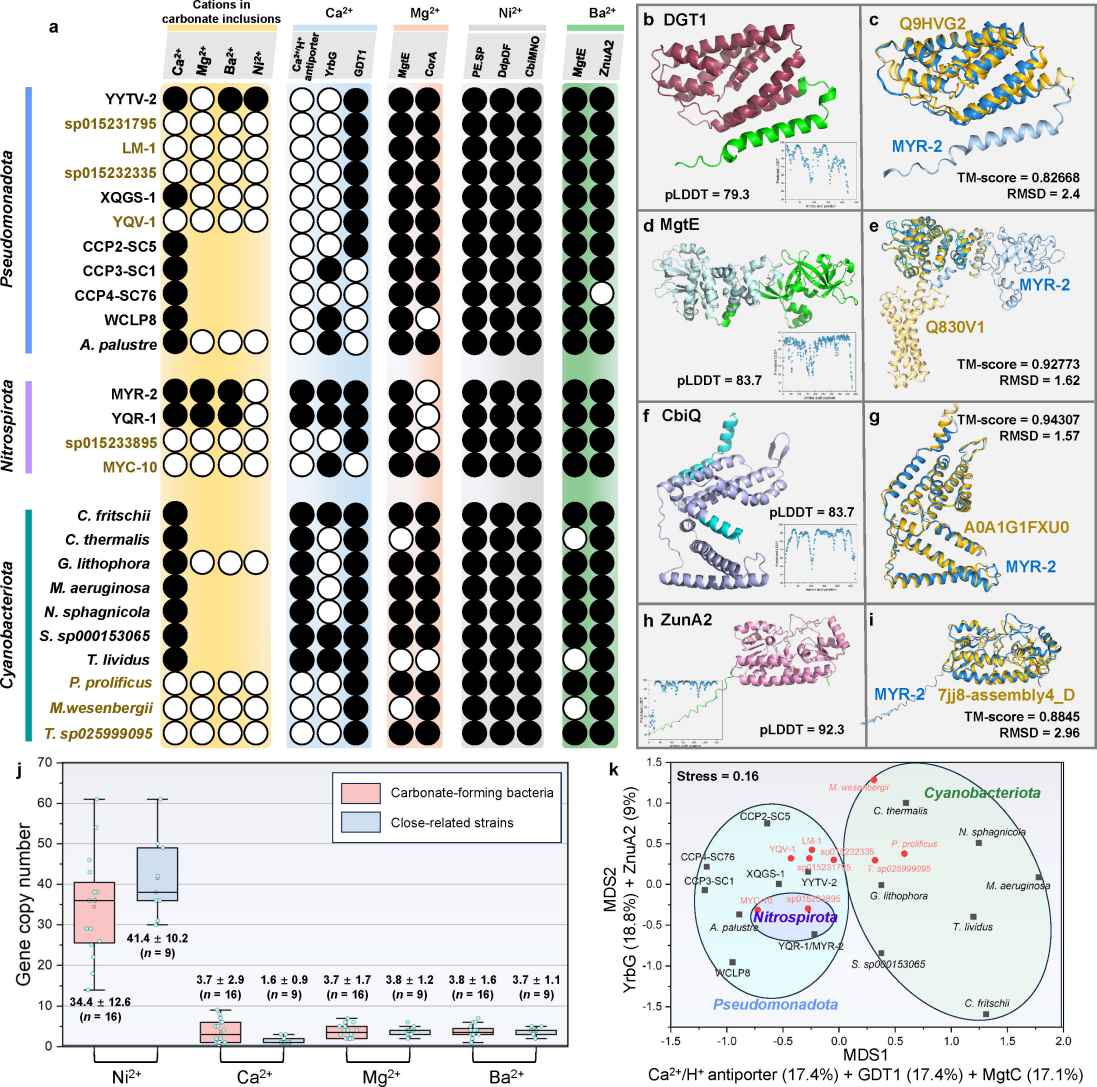
Distributions and predicted structures of Ca^2+^, Mg^2+^, Ni^2+^, and putative Ba^2+^ transporters in intracellular carbonate-forming bacteria and related taxa. (**a**) Presence-absence matrix for cation transporters (rows) and corresponding cation incorporation into intracellular carbonate granules (columns) across carbonate‐forming bacteria (black shading) and closely related organisms (yellow shading). Filled circles denote the confirmed presence (genomic and/or experimental evidence) of a transporter gene or cation in carbonate inclusions. Open circles denote a confirmed absence. Blank cells indicate unavailable data. Transporters GDT1 (Ca²^+^), MgtE/CorA (Mg²^+^), CbiO (Ni²^+^), and ZnuA2 (Zn²^+^/Ba²^+^) are highlighted. (**b–i**) Three‐dimensional structural models of key metal permeases from MYR-2 and YQR-1 (protein sequences are identical in both ecotypes; TM‐scores > 0.82 relative to known permeases). Insets are predicted local-distance difference test (pLDDT) confidence plots (x-axis: residue position; y-axis: pLDDT score). (**b**) Predicted structure of the Ca²^+^ transporter GDT1. The GDT1 domain is highlighted in crimson. (**c**) Superposition of MYR-2 GDT1 (blue) with its top Foldseek template (gold). Root‐mean‐square deviation (RMSD) and TM-score indicate structural conservation. (**d**) Predicted structure of the Mg²^+^ transporter MgtE. The MgtE domain is highlighted in cyan. (**e**) Overlay of MYR-2 MgtE (blue) with the best Foldseek match (gold). (**f**) Predicted structure of the Ni²^+^ transporter CbiO. The EcfT‐like domain is highlighted in blue. (**g**) Structural alignment of MYR-2 CbiO (blue) and its Foldseek hit (gold). (**h**) Predicted structure of the Zn²^+^/Ba²^+^ transporter ZnuA2. The TroA-like domain is highlighted in carmine. (**i**) Superimposition of MYR-2 ZnuA2 (blue) with its closest Foldseek homolog (gold). (**j**) Copy numbers of metal permease genes in carbonate-forming bacteria and closely related non-calcifying strains. (**k**) Non-metric multidimensional scaling (nMDS) and envfit analyses based on the normalized copy numbers of individual transporter genes.

We recovered high-quality draft genomes for strains YQR-1, MYR-2, and YYTV-2 (97.7%–99.3% completeness, ≤0.91% contamination), and a medium-quality genome for XQGS-1 (81.7% completeness, 2.16% contamination) (Data S3) (21). BLAST searches against these metagenome-assembled genomes (MAGs) identified genes encoding multiple metal permease systems in all calcifying ecotypes: the Ca²^+^ transporter GDT1, the Mg²^+^ transporter MgtE or CorA, the Ni²^+^ transporter CbiO, and the Zn²^+^/Ba²^+^ transporter ZnuA2 (Fig. 4a; Data S3 to S8). Notably, MgtE (KO: K15122) functions as both an established Mg²^+^ transporter and a potential facilitator of Ba²^+^ transport (22). Each putative transporter shares ≥30% sequence identity, ≥70% coverage, and an E-value ≤10^−5^ with characterized homologs.

Structural modeling further reveals that these transporters contain canonical cation-binding domains (i.e., Gdt1, MgtE, EcCorA-like, EcfT, and TroA-like) and share high structural similarity (TM-score >0.82 and root-mean-square deviation [RMSD] <3) with characterized metal permeases (Fig. 4b through 4i; Fig. S10 to S13; Data S9). Although bivalent cation transporter homologs are distributed broadly in both calcifying and non-calcifying bacteria (Fig. 4a; Data S8), comparative genomic analyses reveal that the copy number of Ca²^+^ transporters in calcifying bacteria (3.7 ± 2.9, *n* = 16) is significantly higher than in non-calcifying bacteria (1.6 ± 0.9, *n* = 9) (Fig. 4g; Data S8). Non-metric multidimensional scaling (nMDS) and envfit analyses based on the copy numbers of metal permeases (Fig. 4h) reveal that (i) no significant differentiation is observed between calcifying and non-calcifying cyanobacteria, possibly because cyanobacteria primarily regulate Ca²^+^ homeostasis via the calcyanin protein (17); (ii) calcifying and non-calcifying strains of *Pseudomonadota* and *Nitrospirota* are distinguishable along the MDS1 and MDS2 axes, respectively; (iii) the MDS1 axis is associated with the copy numbers of Ca²^+^/H^+^ antiporters (17.4%), GDT1 (17.4%), and MgtC (17.1%), while MDS2 is associated with YrbG (18.8%) and ZnuA2 (9%). These findings suggest that the copy numbers of metal transporter genes may correlate with calcification traits, further supporting the idea that metal transporters play a role in intracellular calcification.

## DISCUSSION

Intracellular calcification in bacteria was first observed in *Achromatium* sp. as early as 1892 (23), and has since been documented in various freshwater, acidic lake, and salt marsh habitats (13, 14, 24–27). More recently, numerous cyanobacterial species have also been shown to form intracellular amorphous calcium carbonate inclusions (15–17, 28) under diverse conditions, ranging from soils, hot springs, freshwater, and seawater (17). In contrast, intracellular calcification in MTB has only recently been recognized. Two alphaproteobacterial strains, CCP-1 from Lake Pavin (France) and XQGS-1 from Xingqinggong Lake (China), are intracellular calcifiers (18, 19). Follow-up work at Lake Pavin further revealed multiple calcifying MTB strains affiliated with four *Gammaproteobacteria* and *Alphaproteobacteria* families (20).

We investigated three MTB strains (YYTV-2, MYR-2, and YQR-1) isolated from freshwater lakes in eastern China (Yuyuantan, Miyun, and Yuqiao Lakes) using both phylogenetic and mineralogical analyses. These strains, affiliated with evolutionarily distinct phyla (*Pseudomonadota* and *Nitrospirota*), all produce intracellular carbonate inclusions alongside magnetosomes. This discovery extends the documented range of intracellular calcifiers to the deep-branching *Nitrospirota* phylum, which supports the hypothesis that biocalcification may have an ancient evolutionary origin (17). It also raises the possibility that intracellular biocalcification may be more phylogenetically and ecologically widespread than recognized. To better quantify its ecological significance, future mineralogical surveys should target not only freshwater systems but also saline, marine, soil, and extreme environments (13, 14, 16).

Collectively, existing evidence suggests that intracellular calcifying bacteria span a broad phylogeny and global distribution, playing significant roles in carbon cycling via processes, such as CO_2_ fixation, in the photic zone (e.g., by cyanobacteria) and OATZs (e.g., by *Achromatium* and MTB) (14, 16, 20). In contrast to carbonate inclusions from *Cyanobacteriota* and *Pseudomonadota* that commonly incorporate only Ca (16–18, 20, 28–30) or Ca/Mg/Ba/Sr (15, 19), our analyses reveal that diverse MTB from the *Pseudomonadota* and the *Nitrospirota* phyla can form carbonates enriched in Ni²^+^, as well as Ca, Mg, and Ba. In YYTV-2 cells, however, only a subset of carbonate inclusions contains detectable Ba²^+^ and Ni²^+^, which suggests heterogeneous metal incorporation (Table S1). This variability may reflect dynamic biological regulation of cation enrichment at the single-cell level, or fluctuations in local metal availability in the surrounding microenvironment (31, 32). Such heterogeneity is consistent with a biologically controlled, environmentally modulated biomineralization process. Overall, this chemical diversity underscores the potential contribution of MTB to heavy metal cycling, particularly for Ba²^+^ and Ni²^+^, in aquatic OATZ environments.

In summary, we describe three MTB ecotypes (MYR-2, YQR-1, and YYTV-2), which span the *Pseudomonadota* and *Nitrospirota* phyla, that concomitantly biomineralize magnetite-type magnetosomes and intracellular Ca–Ba–Mg–Ni carbonate inclusions. Extending intracellular calcification to a deep-branching *Nitrospirota* lineage suggests that such biocalcification may be more widespread among bacteria than previously understood. Genomic and structural analyses of metal permeases support a model in which MTB selectively accumulates trace cations to facilitate carbonate formation. Collectively, these findings suggest novel microbial strategies for heavy-metal biogeochemical cycling (2, 10) and broaden our understanding of prokaryotic intracellular biomineralization.

## MATERIALS AND METHODS

### Field sampling, MTB collection, and sample preparation

Surface sediments were collected from three freshwater lakes in Beijing and Tianjin, eastern China (Table 1). Microcosms simulating the oxic-anoxic transition zone (OATZ) were constructed following Li et al. (33). MTB were extracted magnetically from ~150 mL of sediment using a homemade magnetic separation apparatus (34). Cells were washed, concentrated into ~200 µL of Milli-Q water, and subjected to TEM observations, 16S rRNA gene sequencing, metagenome sequencing, and correlative FISH-SEM (33, 34). TEM samples were stored under a pure nitrogen atmosphere at 20°C.

### Aqueous chemistry analysis

To characterize the chemical environment inhabited by MTB, we measured Ca^2+^, Mg^2+^, Ni^2+^, and Ba^2+^ concentrations from sediment pore water of Yuqiao Lake, Miyun Lake, Yuyuantan Lake, and Xingqinggong Lake. The pore waters were acquired by centrifuging the sediment at 6,000 rpm for 30 minutes. Approximately 150 mL of water was filtered through a 0.45 µm membrane and analyzed via inductively coupled plasma mass spectrometry (ICP-MS; Agilent 8900, Agilent Technologies, USA). Detection limits were 0.02 ppm for Ca^2+^ and Mg^2+^, 0.01 ppm for Ba^2+^, and 0.007 ppm for Ni^2+^.

### Molecular and correlative FISH-SEM experiments

16S rRNA genes of MYR-2 and YYTV-2 were amplified using universal bacterial primers 27F/1492R (35). The amplified DNA was purified with a QIAquick gel extraction kit (Qiagen, Germany), ligated using the pMD19-T vector (TaKaRa, Japan), and then cloned into *Escherichia coli* strain DH5α competent cells (Huada Genome Center, Beijing, China). Thirty clones were picked randomly and sequenced using the primer pair of pMD19-T vector, M13F-47 and M13R-48 (Huada Genome Center, Beijing, China). After discarding sequences shorter than 1,100 bp, the remaining sequences were clustered into several operational taxonomic units with cd-hit software (36) using a threshold of 98.7% sequence identity (species criterion level) (37). Remaining sequences were evaluated and corrected for erroneous bases using the Gblocks 0.9B online algorithm with default parameters (38).

Phylogenetic and morphological identification of MYR-2 and YYTV-2 cells was performed by FISH-SEM (34). Specific oligonucleotide probes, YQR1-1423 (5′-TGCACATGTATTGCTACATGTACA-3′, with melting temperature of 58°C, labeled with Cy3, and a universal bacterial probe EUB338 labeled with fluorescein phosphoramidite FAM) and YYTV2-920 (5′-AAACCATCTCTGGTAACCGCC-3′, with melting temperature of 64°C), were used (21). An appropriate number of *E. coli* cells was added as internal control cells. Subsequent epifluorescence microscopy experiments were performed using an Olympus BX51 microscope. The same sample was then carbon-coated using a Leica ACE200 low-vacuum sputter coater (Leica Microsystems, Wetzlar, Germany) and observed with a Zeiss Ultra-55 field emission SEM instrument (Carl Zeiss, Germany) with a working voltage of 5 kV and ∼5 mm working distance.

### Microscopy and microspectroscopy analyses

Conventional TEM observations and SAED were performed on a JEM-F200 (JEOL, Tokyo, Japan) operated at 200 kV. High-angle annular dark-field HAADF-STEM analysis and EDXS mapping were conducted on a JEM-2100F (JEOL, Tokyo, Japan) at 200 kV equipped with an X-Max detector (Oxford Instruments). EELS data were acquired at C K-edge (280–320 eV) for cytoplasm, Ca *L*_2,3_-edge (340–360 eV) and Ba *M*_4,5_-edge (760–810 eV) for carbonate inclusions, and Fe *L*_2,3_-edge (700–730 eV) for magnetosomes. Semi-quantitative STEM-EDXS spectra were measured using the JEOL Analysis Station with *K*-factor corrections (39).

### Metagenome sequencing, scaffold assembly, and genome binning

Genomic DNA from magnetically concentrated MYR-2, YYTV-2, and XQGS-1 cells was amplified using the REPLI-g Single Cell Kit (Qiagen, Germany). Purified DNA was sequenced on an Illumina HiSeq 6000 (150 bp paired-end reads, insert size ~270 bp) (Annoroad, Beijing, China). Reads were trimmed to remove adapter sequences and low-quality bases using the Trimmomatic software (version 0.39) (40). Clean reads were assembled into scaffolds using the Megahit software (version 1.2.8) (41) with kmer = 39, 59, 79, 99, 119,141, respectively, and optimal scaffolds assembled with different kmer values were filtered. Scaffolds were binned and reassembled with MetaWRAP (version 1.3) (42); scaffolds shorter than 1,500 bp were abandoned. Genome completeness and contamination were evaluated using CheckM2 (version 1.0.2) (43), which yielded 97.7%–99.3% completeness estimates with ≤0.91% contamination for MYR-2 and YYTV-2, and 81.7% completeness with 2.16% contamination for XQGS-1. These values are within accepted thresholds for reliable recovery of subsequent functional genes from metagenome-assembled genomes (44). Average nucleotide identity was calculated with pyani (version 0.2.9) (https://github.com/widdowquinn/pyani).

### Phylogenetic analyses

Genome taxonomy was assessed using GTDB-Tk (v2.4.0) with the GTDB database (r220) (45). Two phylogenetic trees for the *Rhodospirillales* order (rooted by the *Azospirillaceae* family) and the *Thermodesulfovibrionia* class (rooted by the *Nitrospirales* order) were subsequently constructed. High-quality genomes (completeness ≥90% and contamination ≤5%) were filtered from each genus of the *Rhodospirillales* order and the *Thermodesulfovibrionia* class based on the GTDB R220 database. The above reference genomes are summarized in Data S1 and S2. Their multiple alignments of 120 single-copy bacterial marker protein sequences were constructed using the GTDB-Tk (v2.4.0) “classify_wf” command. Maximum-likelihood trees were built using IQ-Tree (version 2.2.2.6) (46) with models selected by ModelFinder (47) and 1,000 ultrafast bootstrap replicates. Trees were visualized using FigTree v1.4.4 (http://tree.bio.ed.ac.uk/software/figtree/).

### Protein functional prediction and 3D structure modeling

Due to a lack of specialized Ba^2+^ transporters, permeases with broad substrates, including Ba^2+^ (i.e., heavy metal permease ZnuA2 [48], Mg^2+^ transporter MgtE [22], and citrate transporter CitH [49]), are used as potential Ba^2+^ channels. Ca^2+^, Mg^2+^, Ni^2+^, and potential Ba^2+^ transporters in the Kyoto Encyclopedia of Genes and Genomes (KEGG) database (50) are summarized in Extended Data 4. All of their protein sequences were downloaded from the Annotree (51) and Uniprot databases (52) according to KEGG entry number and are summarized in Data S5 and S6 as BLAST subject sequences. BLAST searches were conducted using the offline BLAST package (53) with the following criteria: ≥30% identity, ≥70% subject coverage, ≥70% query coverage, and E-value ≤10^−5^. All BLAST results are summarized in Data S7.

Conserved protein domains based on amino acid sequences were analyzed using the CDD database (54). 3-D protein structure models were constructed using the DeepMind AlphaFold2 software (55) and visualized with the PyMOL software (version 2.4). Predicted local-distance difference test (pLDDT) scores were used to assess confidence measures of 3-D protein structure models. Protein structure comparison and conserved domain annotation based on 3D protein structure were performed with the Foldseek online database (56). RMSD and TM-score for evaluating structural similarities were calculated by the Foldseek online database (56).

### Biostatistical analyses

nMDS and envfit analyses were performed based on the copy numbers of nine gene orthologs (K02034, K02008, K07301, K23541, K07507, K03284, K15122, K09815, and the Ca²^+^/H^+^ antiporter) that are conserved in most carbonate-forming bacteria. These analyses were conducted using the Vegan package in R, which provides community ecology analysis functions. nMDS is a rank-based ordination method in which original Euclidean distances are replaced by their ranked values. Gene copy numbers were normalized by dividing each count by the maximum value observed across all tested strains. Subsequently, envfit was applied to fit gene copy numbers onto the nMDS ordination to evaluate the influence of each transporter on the distribution of calcifying versus non-calcifying bacteria. Genes with R² >0.5 and *P* ≤0.01 are considered to be correlated significantly with the calcification trait.

## Data Availability

The genome sequences of MYR-2, YYTV-2, and XQGS-1 have been deposited in the NCBI database under accession numbers JBHMJA000000000, JBHMJB000000000, and JBKACM000000000 within BioProject PRJNA657227. The 16S rRNA gene sequence of MYR-2 and YYTV-2 is under accession numbers PP905514 and PP907732.

## SUPPLEMENTAL MATERIAL

Supplemental figures and table msystems.01131-25-s0001.pdf Figures S1 to S13 and Table S1.

Supplemental material msystems.01131-25-s0002.xlsx Data S1 to S9.

ASM does not own the copyrights to Supplemental Material that may be linked to, or accessed through, an article. The authors have granted ASM a non-exclusive, world-wide license to publish the Supplemental Material files. Please contact the corresponding author directly for reuse.
