# Supplementary material for: Deep-branching magnetotactic bacteria form intracellular carbonates enriched in trace metals
Source: mSystems. 2025 Nov 12;10(12):e01131-25. doi: 10.1128/msystems.01131-25 (PMC12710302; doi:10.1128/msystems.01131-25)
Supplement: Supplemental figures and table — Figures S1 to S13 and Table S1. [file msystems.01131-25-s0001.pdf]

Supplementary Materials for

Deep-branching magnetotactic bacteria form intracellular carbonates enriched in trace metals

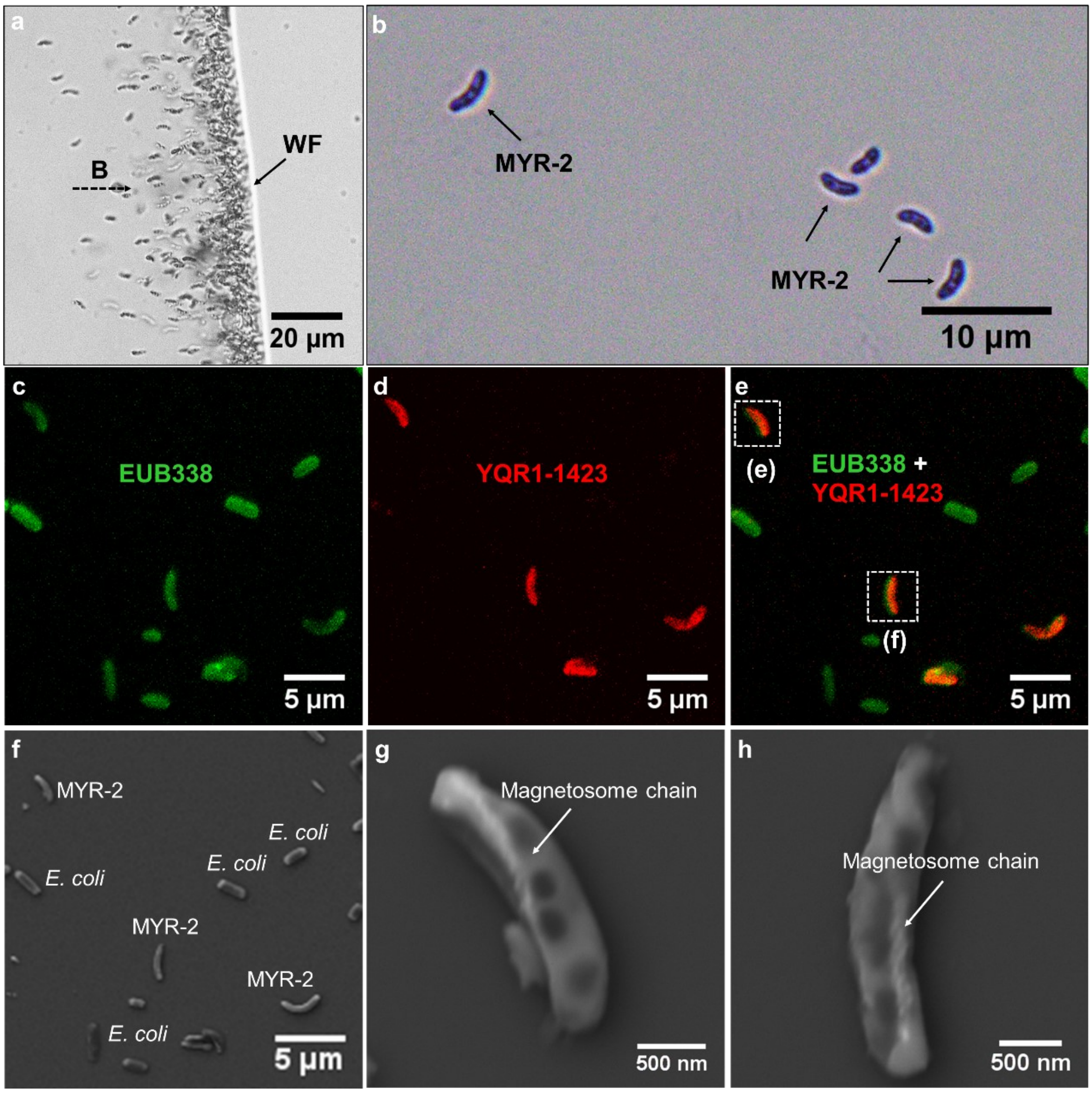

Fig. S1 Morphological and phylogenetic identification of strain MYR-2 cells using a correlative FISH-SEM approach. (a) Optical micrograph of living MTB cells swimming parallel to an applied magnetic field B and gathering at a droplet water front (WF). (b) Optical micrograph of magnetically collected bacteria stained with crystal violet. MYR-2 cells are indicated by solid black arrows. (c) Fluorescence micrograph of bacteria hybridized *in situ* with the 5′-FAM-labeled universal bacterial probe EUB338. (d) Fluorescence micrograph of bacteria hybridized *in situ* with the 5′-Cy3-labeled YQR-1-specific probe YQR1-1423. (e) Overlapped fluorescence microscopy image of (c) and (d). (f) Coordinated SEM image of the same field of view as in (e). (g) and (h)**,** Close-up of the bacteria indicated by the dashed-line boxes in (e).

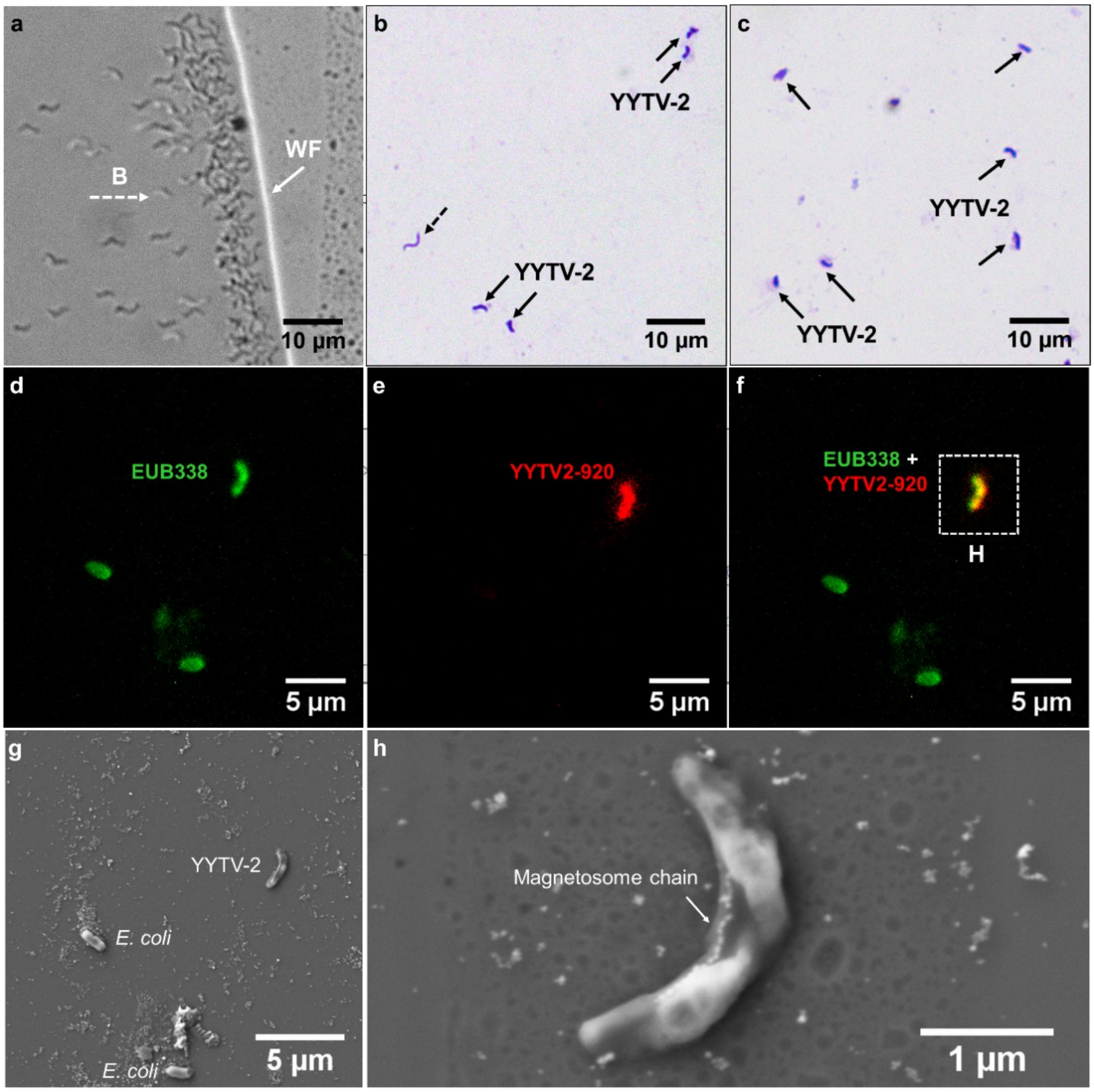

Fig. S2 Morphological and phylogenetic identification of strain YYTV-2 cells using a correlative FISH-SEM approach. (a) Optical micrograph of living MTB cells swimming parallel to an applied magnetic field B and gathering at a droplet water front (WF). (b) and (c) Optical micrograph of magnetically collected bacteria stained with crystal violet. YYTV-2 cells are indicated by solid black arrows; unknown spirilla are indicated by dashed black arrows. (d) Fluorescence micrograph of bacteria hybridized *in situ* with the 5′-FAM-labeled universal bacterial probe EUB338. (e) Fluorescence micrograph of bacteria hybridized *in situ* with the 5′-Cy3-labeled YYTV-2-specific probe YYTV2-920. (f) Overlapped fluorescence microscopy image. (g) Coordinated SEM image of the same field of view as in (f). (h) Close-up of the bacteria indicated by the dashed-line box in (f).

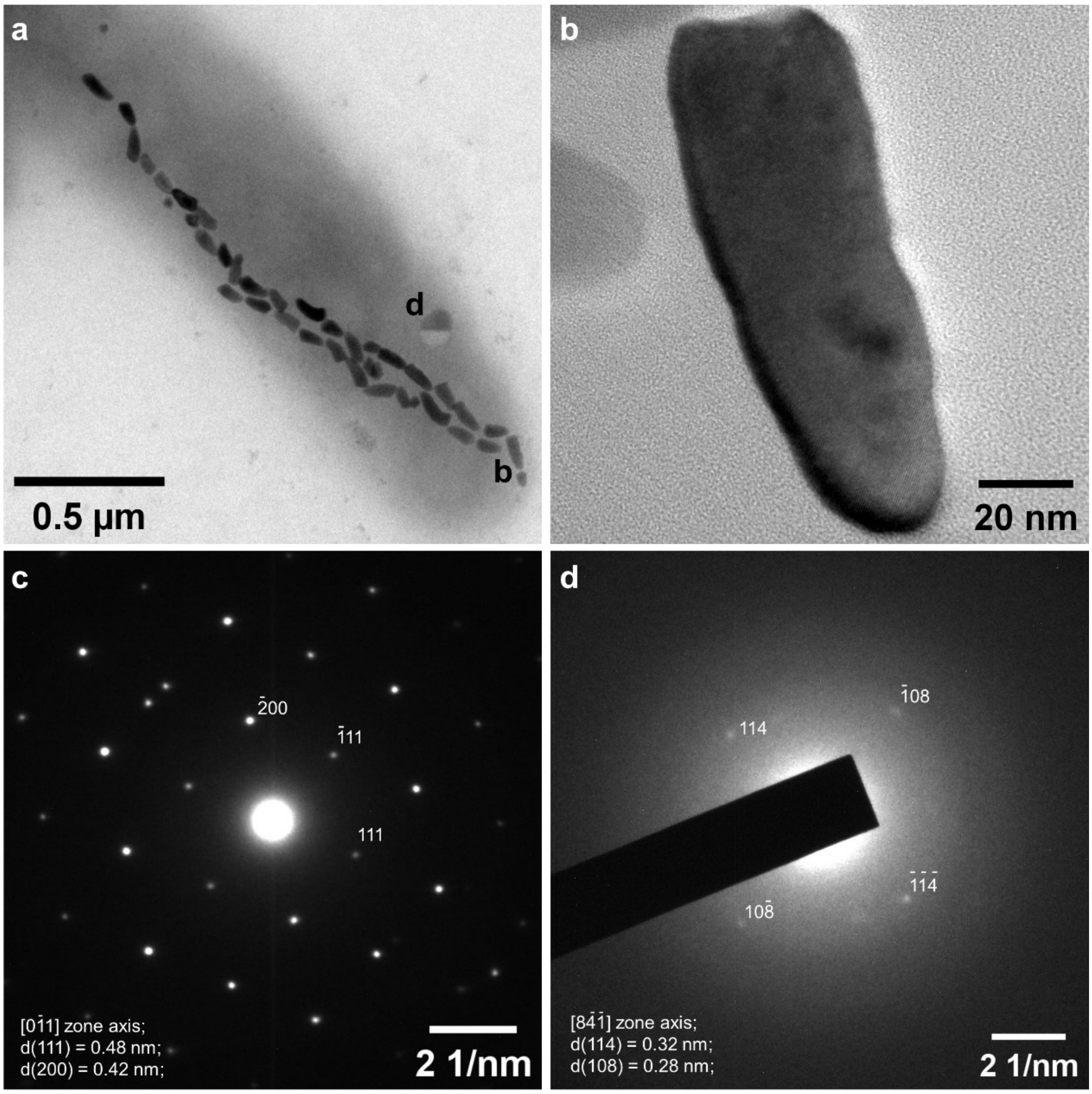

Fig. S3. Morphological and structural features of intracellular inclusions in a MYR-2 cell. (a) TEM image of a MYR-2 cell that contains two electron-dense particle types. (b) - (d) Selected area electron diffraction (SAED) patterns recorded from (b) an individual magnetite particle (labeled 'b' in (a)) and (d) a carbonate granule (labeled 'd' in (a)). SAED analyses confirmed that the magnetic particles are well-crystallized single crystals with d-spacings of 0.48 nm and 0.42 nm, which correspond to the interplanar spacings (d) of the {111} and {200} planes of magnetite, respectively. By contrast, the SAED pattern in (d) has several weak, smeared diffraction rings with d-spacings of 0.32 nm and 0.28 nm, which indicates that the carbonate inclusion is weakly crystalline.

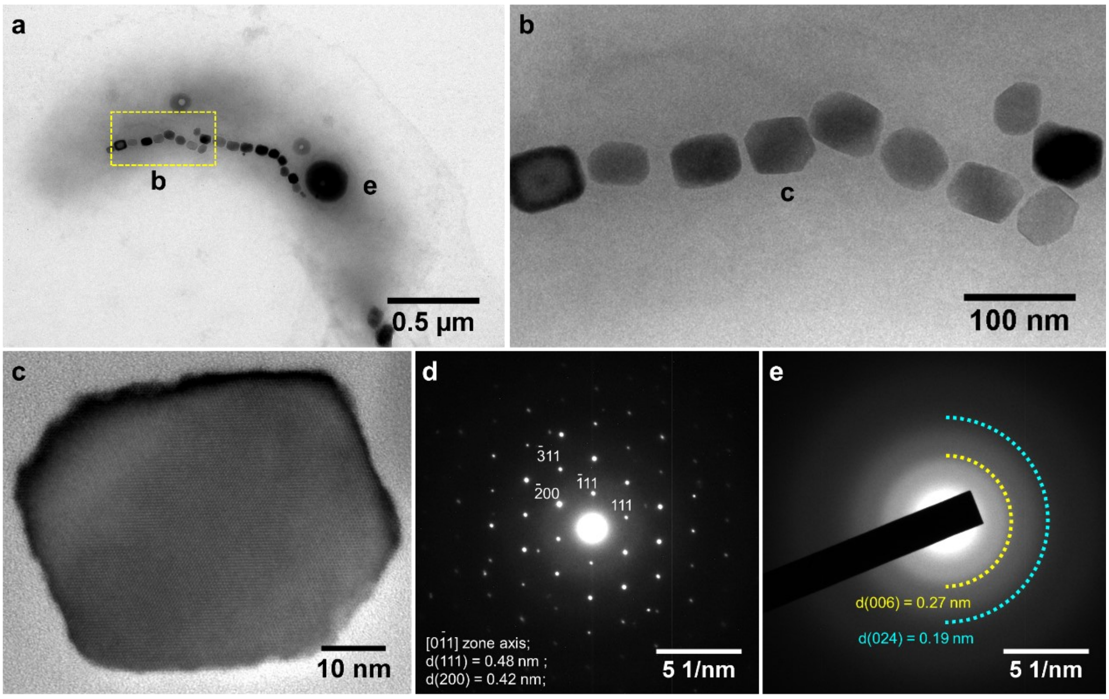

Fig. S4. Morphological and structural features of intracellular inclusions within a YYTV-2 cell. (a) TEM image of a YYTV-2 cell that contains two electron-dense particle types. (b) Close-up of a YYTV-2 magnetosome chain indicated by the yellow dashed box in (a). (c) - (e) Selected area electron diffraction (SAED) patterns recorded from (c) an individual magnetite particle (labeled ‘c’ in (b)) and (d) a carbonate granule (labeled ‘d’ in (a)). SAED analyses indicate that the magnetic particles are well-crystallized single crystals with d-spacings of 0.48 nm and 0.42 nm, which correspond to the interplanar spacings (d) of the {111} and {200} planes of magnetite, respectively. By contrast, the SAED pattern in (e) has two weak, smeared diffraction rings that indicate that carbonate inclusions are amorphous.

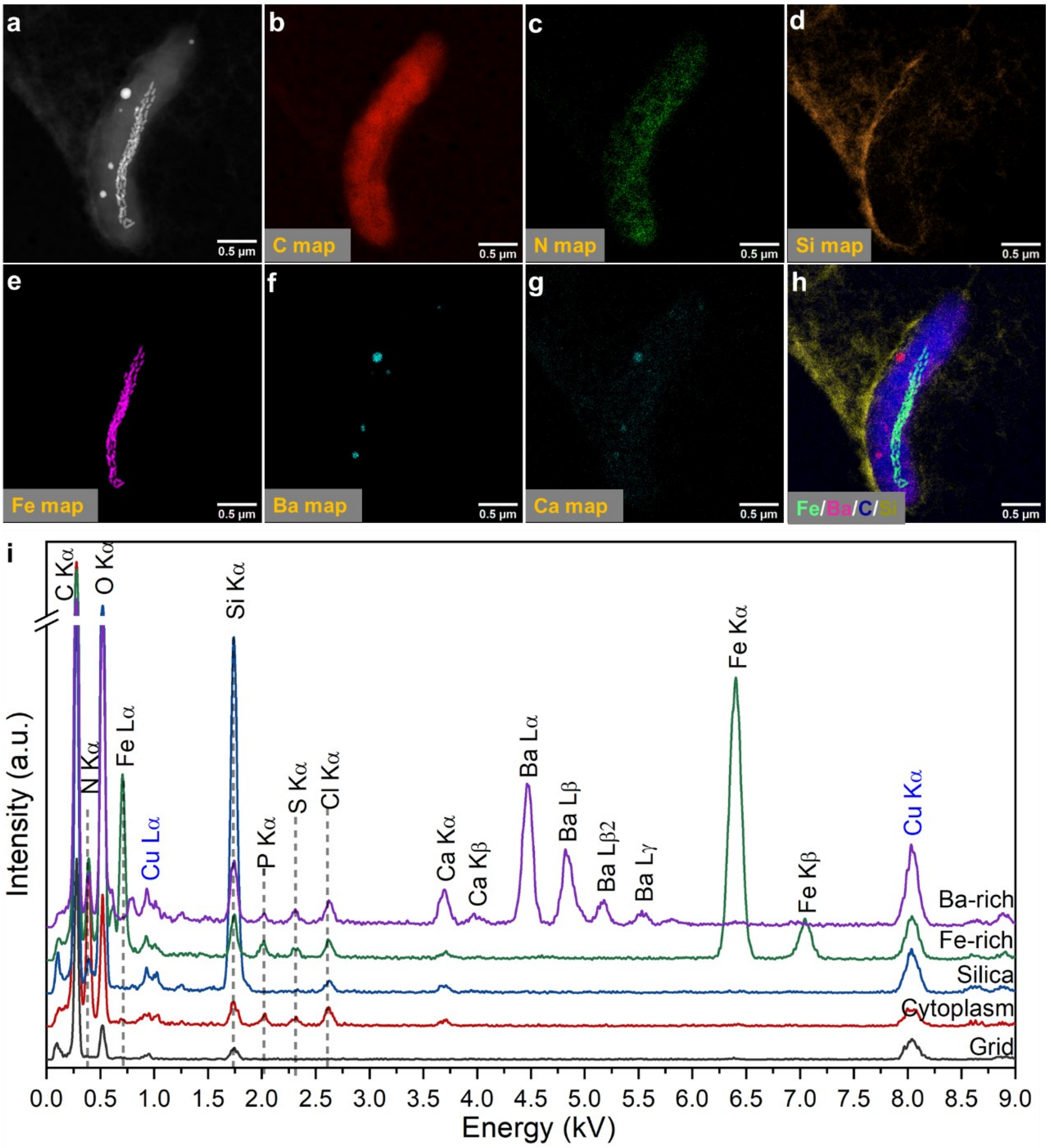

Fig. S5 Chemical characterization of calcium-barium carbonate inclusions within a MYR-2 cell. (a) STEM-HAADF image of a MYR-2 cell with three carbonate inclusions. **b - h,** STEM-EDXS elemental maps of the MYR-2 cell in (a). (b) Carbon (C Kα emission line). (c) Nitrogen (N Kα). (d) Silicon (Si Kα). (e) Iron (Fe Kα). (f) Barium (Ba Lα). (g) Calcium (Ca Kα). (h) RGB map of C (blue), Fe (green), Si (yellow), and Ba (red). (i) STEM-EDX spectra from different regions of interest: carbonate inclusion, magnetosome, silicon-rich floccule outside the MYR-2 cell, cytoplasm, and TEM grid. a.u., arbitrary units.

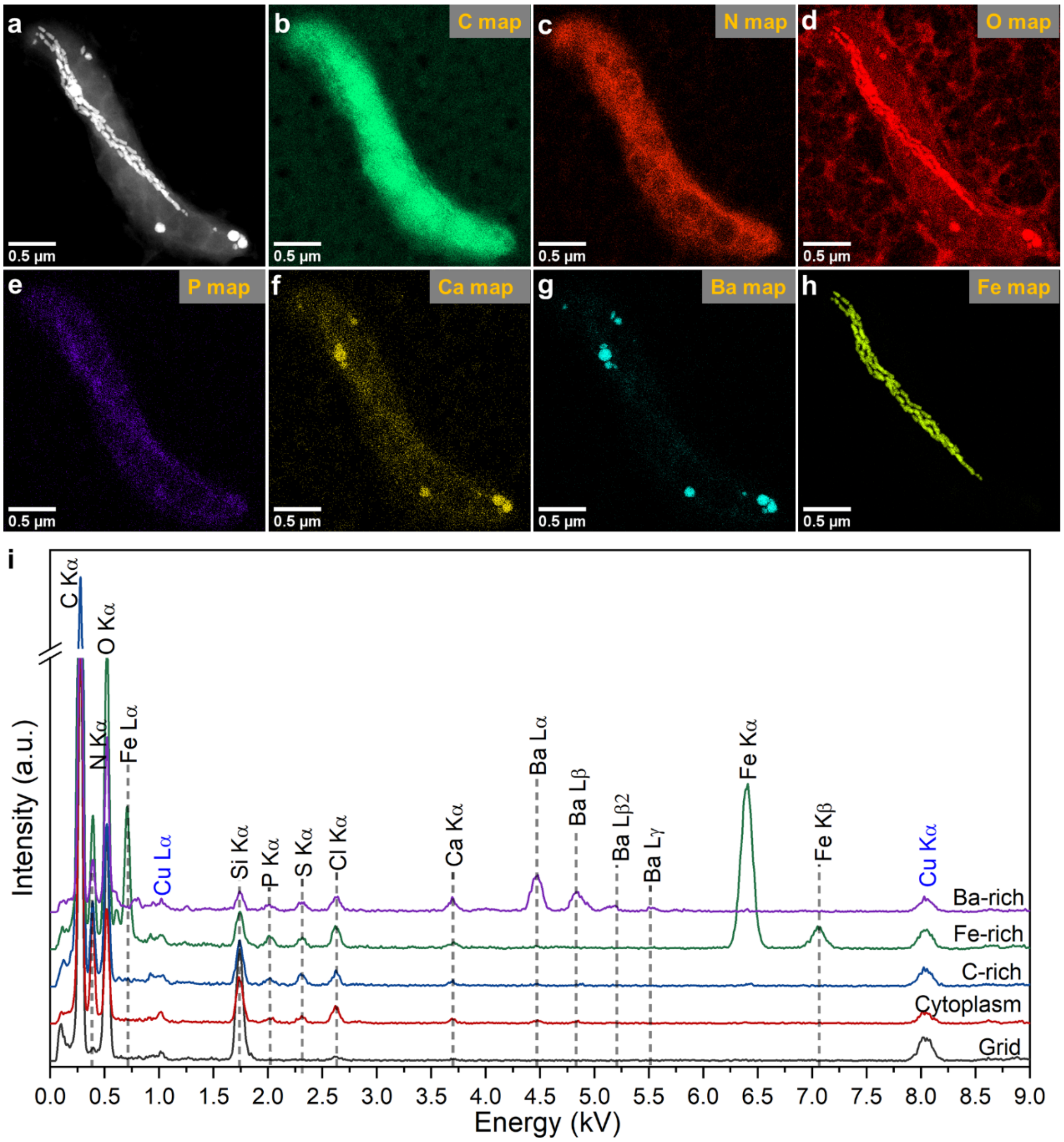

Fig. S6 Chemical characterization of calcium-barium carbonate inclusions within a MYR-2 cell. (a) STEM-HAADF image of a MYR-2 cell with several carbonate inclusions. (b) – (h) STEM-EDXS elemental maps of the MYR-2 cell in (a). (b) Carbon (C Kα). (c) Nitrogen (N Kα). (d) Oxygen (O Kα). (e) Phosphorus (P Kα). (f) Calcium (Ca Kα). (g) Barium (Ba Lα). (h) Iron (Fe Kα). (i) STEM-EDX spectra from different regions of interest: carbonate inclusion, magnetosome, carbon-rich globule, cytoplasm, and TEM grid. a.u., arbitrary units.

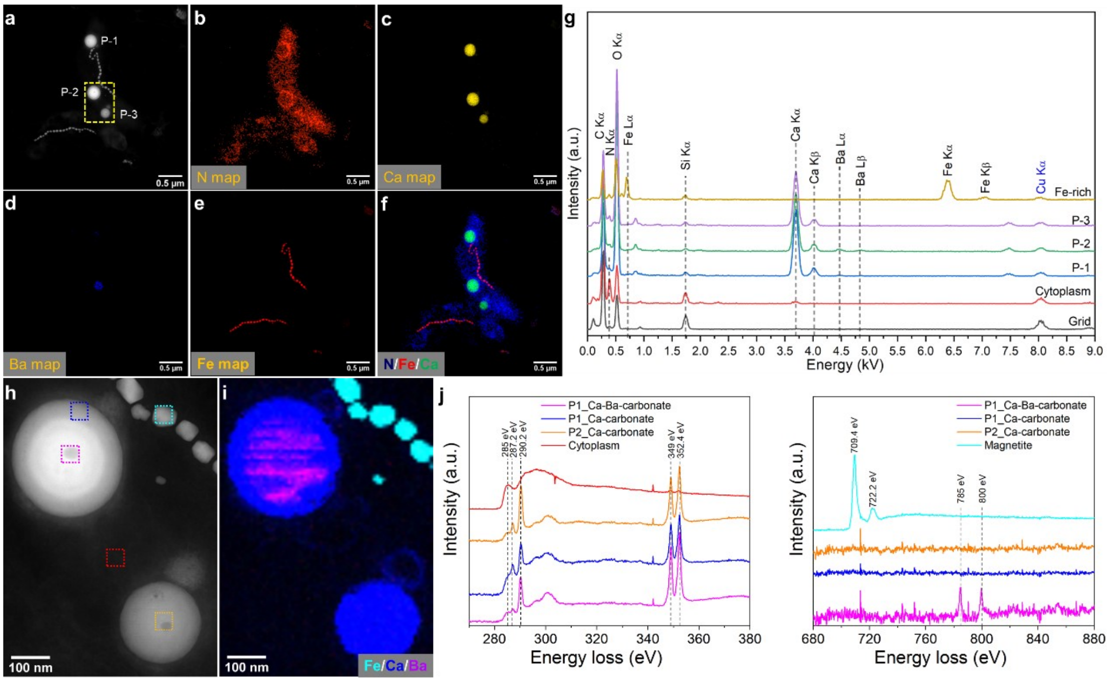

Fig. S7 Chemical and mineralogical characterization of calcium-barium carbonate inclusions within MTB strain YYTV-2 cells. (a) STEM-HAADF image of two YYTV-2 cells. (b) – (f) STEM-EDXS elemental maps of YYTV-2 cells in (a). (b) Nitrogen (N Kα). (c) Calcium (Ca Kα). (d) Barium (Ba Lα). (e) Iron (Fe Kα). (f) Red-green-blue (RGB) map of N (blue), Fe (red), and Ca (green). (g) STEM-EDX spectra from different regions of interest: magnetosome (Fe-rich), carbonate inclusions (P-1, P-2, and P-3), cytoplasm, and TEM grid. a.u., arbitrary units. (h) HRTEM image of two carbonate inclusions in YYTV-2 cells within the yellow dashed box in (a). (i) RGB map of Ca (blue), Fe (cyan), and Ba (purple) distributions in carbonate inclusions in (h) based on electron energy loss spectra (EELS). (j) TEM-based EELS analyses at the Fe $L_{2,3}$-edge (700-730 eV), Ca $L_{2,3}$-edge (340-360 eV), and Ba $M_{4,5}$-edge (760-810 eV) on a magnetosome (cyan), Ca-carbonate inclusions (blue and yellow), Ca-Ba carbonate inclusions (carmine), and cytoplasm (red), respectively, within the dashed boxes in corresponding colours in (h). The spectra are identical to those of magnetite, calcium carbonate, and barium carbonate, respectively.

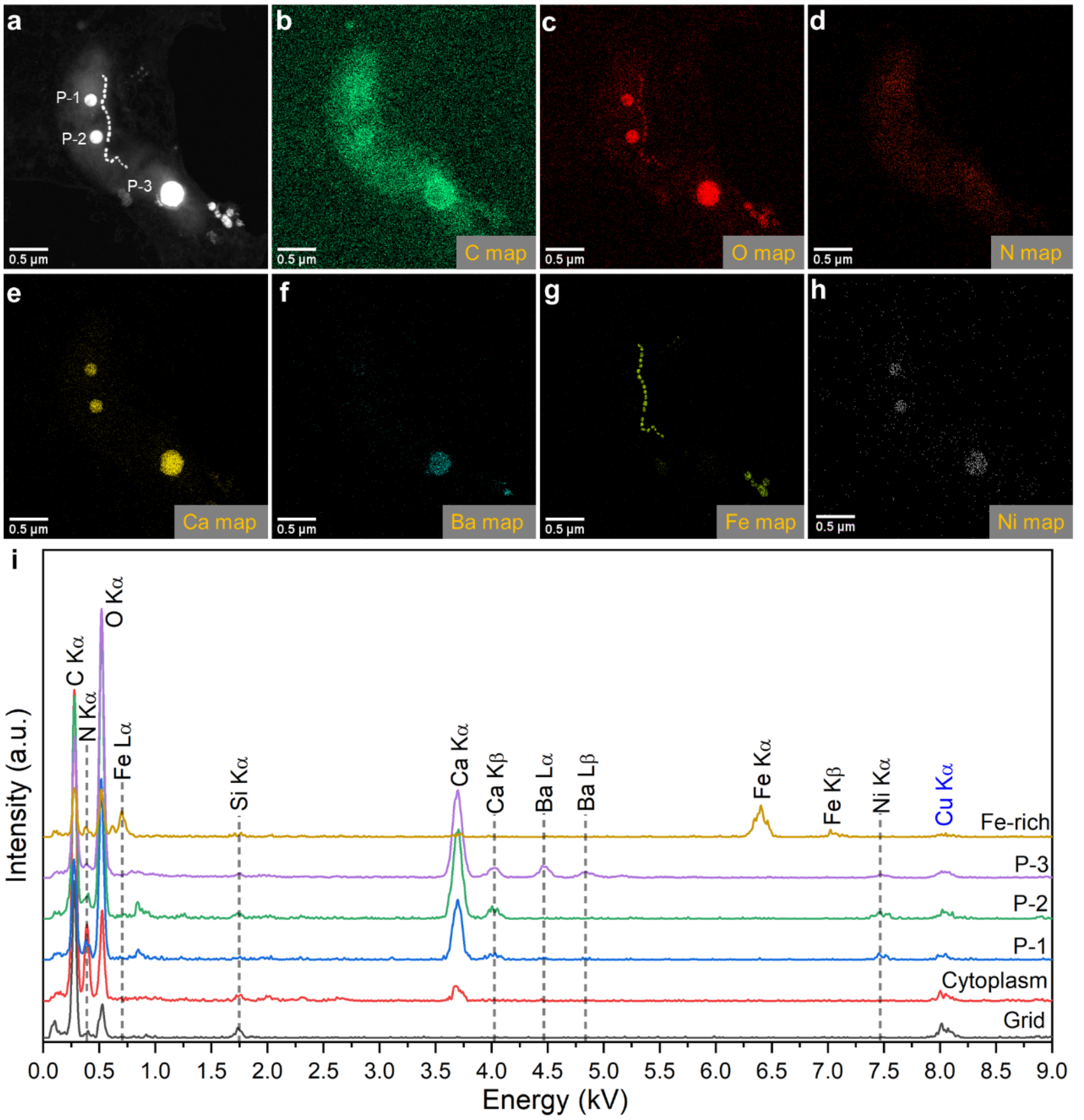

Fig. S8 Chemical characterization of calcium-barium-nickel carbonate inclusions in a YYTV-2 cell. (a) STEM-HAADF image of a YYTV-2 cell. (b) – (h) STEM-EDXS elemental maps of carbonate inclusions in (a). (b) Carbon (C Kα). (c) Oxygen (O Kα). (d) Nitrogen (N Kα). (e) Calcium (Ca Kα). (f) Barium (Ba Lα). (g) Iron (Fe Kα). (h) Nickel (Ni Kα). (i) STEM-EDX spectra from different regions of interest: magnetosome, carbonate inclusion, cytoplasm, and TEM grid. a.u., arbitrary units.

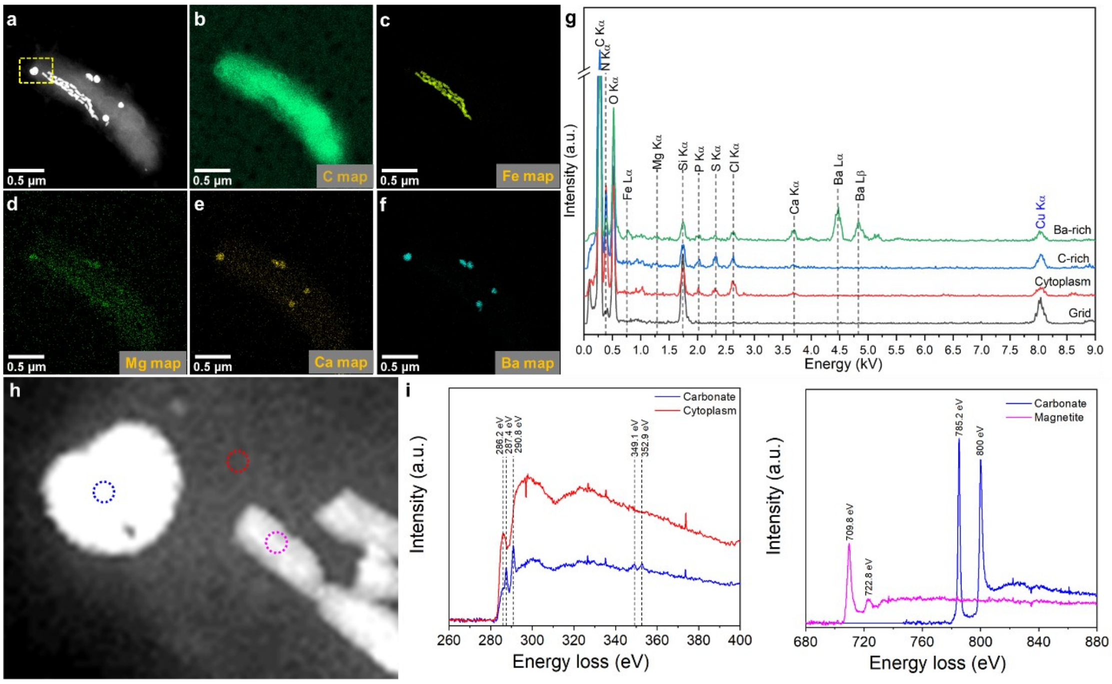

Fig. S9 Chemical and mineralogical characterization of calcium-barium carbonate inclusions within MTB strain YQR-1 cell. (a) STEM-HAADF image of a YQR-1 cell. (b) – (f) STEM-EDXS elemental maps of a YQR-1 cell shown in (a). (b) Carbon (C Kα). (c) Iron (Fe Kα). (d) Magnesium (Mg Kα). (e) Calcium (Ca Kα). (f) Barium (Ba Lα). (g) STEM-EDX spectra for different regions of interest: Ba-rich carbonate inclusion, carbon-rich globule, cytoplasm, and TEM grid. a.u., arbitrary units. (h) HRTEM image of a carbonate inclusion in a YQR-1 cell within the yellow dashed box in (a). (i) TEM-based EELS analyses at the Fe $L_{2,3}$-edge (700-730 eV), Ca $L_{2,3}$-edge (340-360 eV), and Ba $M_{4,5}$-edge (760-810 eV) on a magnetosome (carmine), Ca-Ba carbonate inclusion (blue), and cytoplasm (red), respectively, within the dashed boxes in corresponding colours in (h).

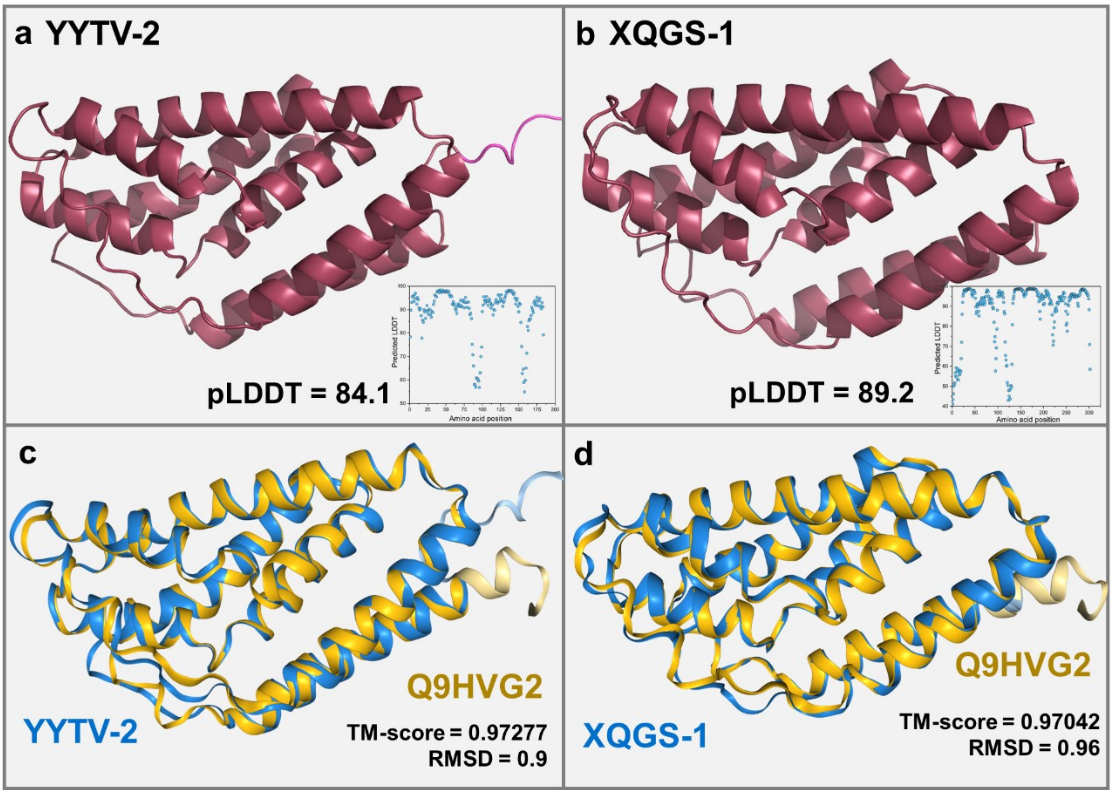

Fig. S10 Modeling of 3-D structures of Ca transporter GDT1 in intracellularly calcifying bacteria. (a) and (b) Homologous proteins to GDT1 in calcareous MTB strains YYTV-2 and XQGS-1. The Gdt1 domain is marked in red. Insets: scatter plots of amino acid confidence. The *x*-axis is amino acid position. The *y*-axis is amino acid pLDDT value. (c) and (d) 3-D structure comparisons of GDT1 protein between YYTV-2, XQGS-1, and their best hits in Foldseek database.

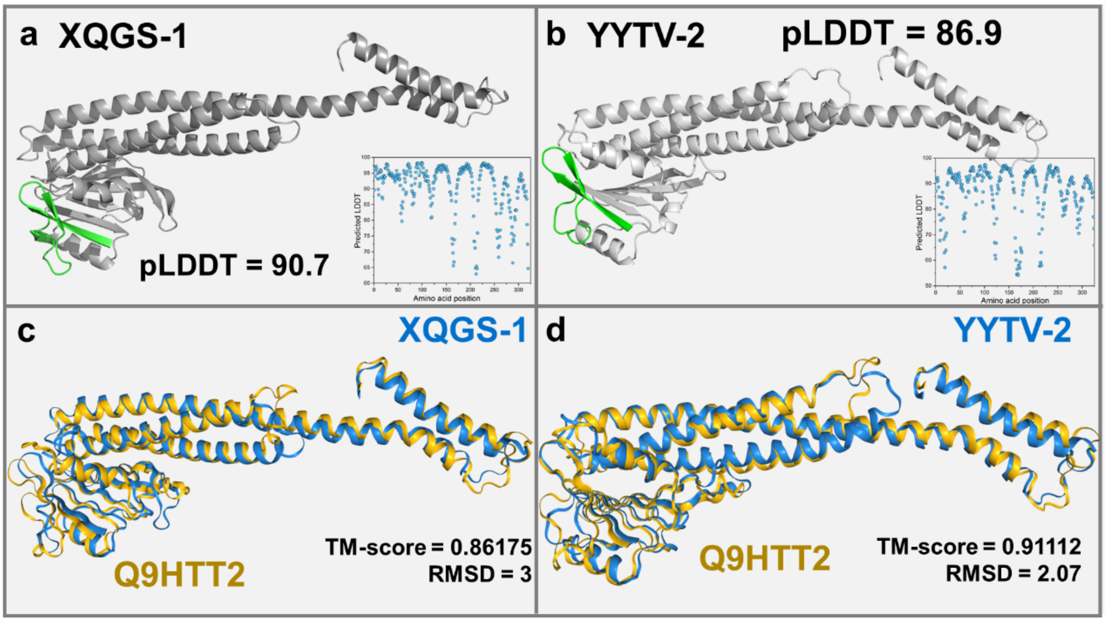

Fig. S11 Modeling of 3-D structures of Mg transporter CorA in intracellularly calcifying bacteria. (a) and (b) Homologous proteins to CorA in calcareous MTB strains XQGS-1 and YYTV-2. The EcCorA-like domain is marked in gray. Insets: scatter plots of amino acid confidence. The *x*-axis is amino acid position. The *y*-axis is amino acid pLDDT value. (c) and (d) 3-D structure comparisons of CorA protein between XQGS-1, YYTV-2, and their best hits in Foldseek database.

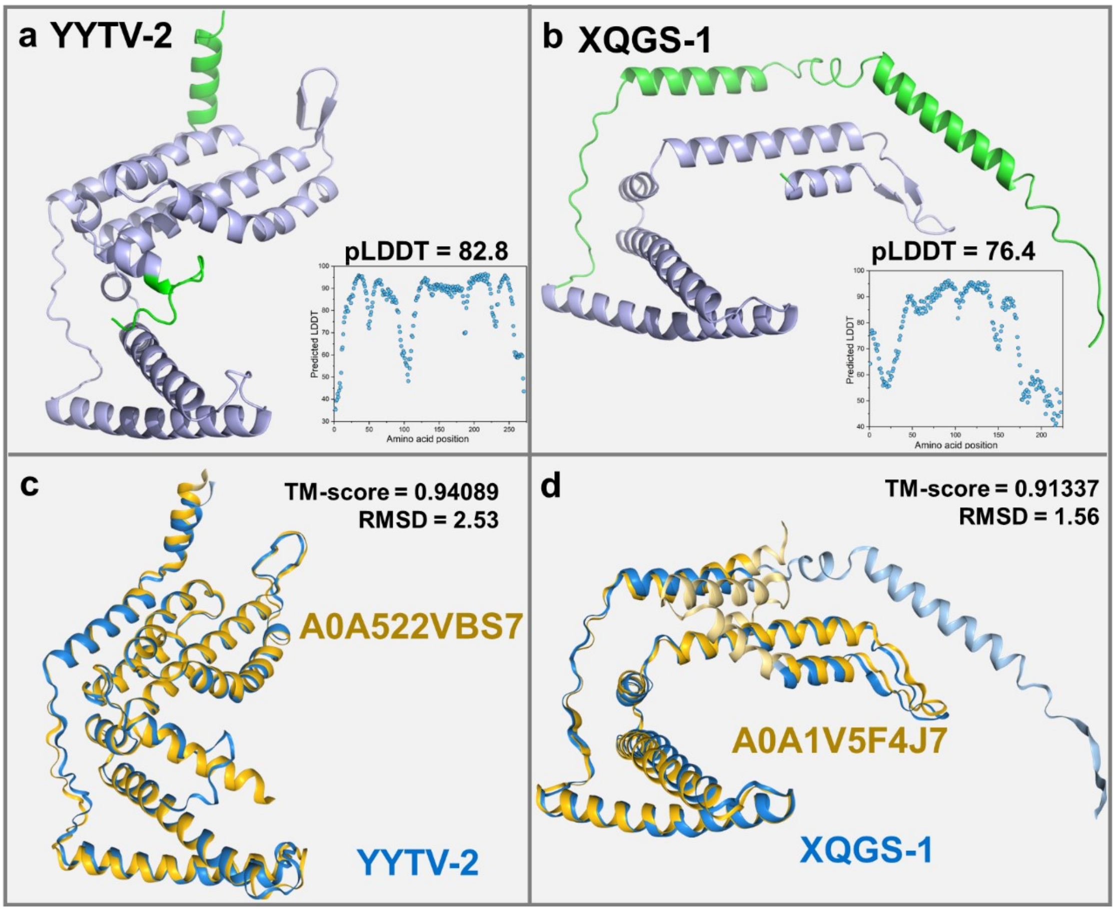

Fig. S12 Modeling of 3-D structures of Ni transporter CbiO in intracellularly calcifying bacteria. (a) and (b) Homologous proteins to CbiO in calcareous MTB strains YYTV-2 and XQGS-1. The EcfT domain is marked in gray. Insets: scatter plots of amino acid confidence. The *x*-axis is amino acid position. The *y*-axis is amino acid pLDDT value. (c) and (d) 3-D structure comparisons of CbiO protein between YYTV-2, XQGS-1, and their best hits in Foldseek database.

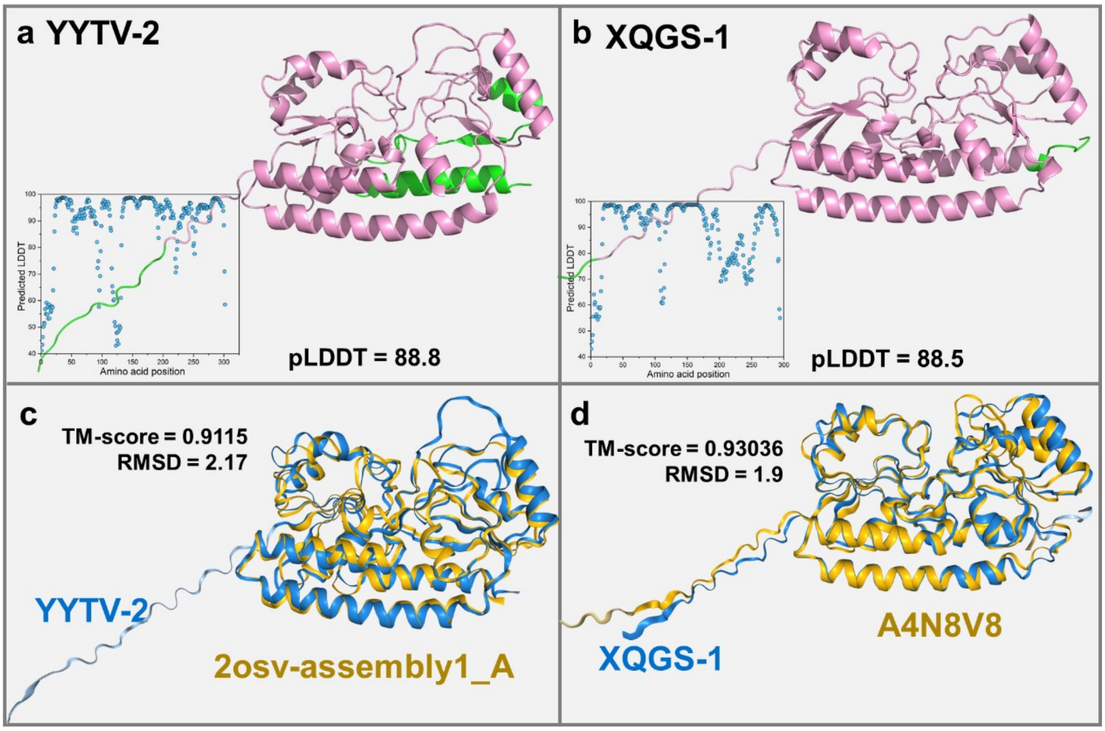

Fig. S13 Modeling of 3-D structures of potential Ba transporter ZnuA2 in intracellularly calcifying bacteria. (a) and (b) Homologous proteins to ZnuA2 in calcareous MTB strains YYTV-2 and XQGS-1. The TroA-like domain is marked in carmine. Insets: scatter plots of amino acid confidence. The *x*-axis is amino acid position. The *y*-axis is amino acid pLDDT value. (c) and (d) 3-D structure comparisons of ZnuA2 protein between YYTV-2, XQGS-1, and their best hits in Foldseek database.

Table S1. Ba/Ca atomic ratios within carbonate-bearing inclusions formed by MTB cells.

Strains	Inclusions	Atomic ratio			
		**Ca K*α***	**Ba L*α***	**Mg K*α***	**Ba/Ca ratio**
YYTV-2	1	97.5	2.3	0.2	0.02
	2	91	8.2	0.8	0.09
	3	99	0	1	0
	4	97.3	0	2.7	0
	5	92.3	6.9	0.8	0.07
	6	88.4	11	0.6	0.12
	7	95.1	5	0.9	0.05
	8	97.2	1.2	1.6	0.01
	9	87.9	11.7	0.4	0.13
	Average	92.5	6.5	1	0.05 ± 0.04
MYR-2	1	9.8	89.3	0.9	9.11
	2	15.6	84.2	0.2	5.4
	3	15.3	84.2	0.5	5.5
	4	10.9	88.7	0.4	8.14
	5	13.4	86.1	0.5	6.43
	Average	13	86.5	0.5	6.92 ± 1.65
YQR-1	1	17.6	78.5	3.9	4.46
	2	4.9	87.5	7.6	17.86
	3	4.1	83.5	12.4	20.37
	4	18	75.9	6.1	4.22
	5	24.3	70.9	4.8	2.91
	6	17.7	76	6.3	4.29
	7	17.4	77.5	5.1	4.46
	Average	14.9	78.5	6.6	8.37 ± 7.4
XQGS-1	1	95.9	--	4.1	--
	2	93.4	--	6.6	--
	3	100	--	0	--
	4	96.2	--	3.8	--
	5	97.5	--	2.5	--
	6	100	--	0	--
	7	100	--	0	--
	8	96.5	--	3.5	--
	9	96.5	--	3.5	--
	10	99	--	1	--
	Average	97.5	--	2.5	--
